# Reducing scheduled evening training does not improve recruits' sleep duration or quality during British Army basic training

**DOI:** 10.3389/fneur.2025.1560518

**Published:** 2025-05-07

**Authors:** Alex J. Rawcliffe, Bethany Moxham, Harry Britt, Katrina Hinde, Shaun Chapman, Andrew Roberts

**Affiliations:** ^1^Army Recruit Health and Performance Research, Medical Branch, HQ Army Recruiting and Initial Training Command, Ministry of Defence, Upavon, United Kingdom; ^2^Human Sciences Group, Defence Science and Technology Laboratory, Salisbury, United Kingdom

**Keywords:** recruits, basic training, sleep duration, sleep quality, military

## Abstract

**Introduction:**

Sleep is critical to the health, wellbeing and performance of recruits during Army basic training, however, is often sacrificed due to the constraints of basic training schedules. In an effort to improve sleep duration of recruits, the revised Common Military Syllabus 21 (CMS21) restricted daily scheduled military training from 18:30, thus enabling greater opportunity for earlier bedtimes and longer nocturnal sleep periods. Our objective was to evaluate the effectiveness of the revised CMS21 basic training programme on measures of sleep-wake indices and compare against the previous programme (CMS18) as a control group.

**Methods:**

Several actigraphy-derived and self-reported sleep-wake indices assessed potential changes in sleep opportunity between groups (CMS21 vs CMS18) and across 12-weeks of basic training. Actigraphy was measured daily to deduce weekly averages and self-report measures (i.e., sleep diaries) were captured during weeks 1, 6 and 11 of basic training. Sleep-wake data are presented descriptively (mean ± SD) and a series of linear-mixed models determined differences in actigraphy between groups and across each week.

**Results:**

Compared to CMS18, no significant improvements in any sleep-wake indices were observed during CMS21. The average sleep duration for both groups remained below the minimum national sleep duration recommendations for young adults (deficit of ~1 h 20 m), with 67% to 94% of recruits in CMS18 and 69% to 97% of recruits in CMS21 achieving an average of <7 h sleep per night, respectively. Similar proportions of recruits reported poor-levels of sleep quality (≤ 60%) during CMS18 and CMS21, with excessive noise and early-morning wake times identified as the most common sleep disturbances. The contracted early-morning feeding times and the magnitude of non-scheduled late-evening military admin were determined as key factors preventing adequate sleep opportunity despite restricting scheduled military training from 18:30 during CMS21.

**Discussion:**

The results of this study warrant the application of improved sleep hygiene practices within the basic training environment. It is also recommended that the contracted early-morning feeding times and magnitude of non-scheduled training activity be considered as factors for change within future programme design to optimize sleep opportunity during basic training.

## Introduction

The primary aim of basic military training is to transform civilians into trained soldiers. Recruits across all trades within the British Army are required to develop and uphold core professional values, master technical skills and improve physical fitness. Failure to meet these standards can result in lower first-time pass rates and jeopardize gains in trained strength. The value and importance of sleep relative to physical and mental health of recruits (1) and trained soldiers (2) is widely recognized as a key priority for success (3). However, despite efforts to improve sleep duration and quality during basic training (4, 5), military culture continues to downplay the importance of sleep, as chronic (i.e., daily) sleep restriction and poor sleep hygiene remain key health-related issues reported in military personnel (1, 4, 6, 7). Military culture presents a significant barrier to promoting healthy sleep practices, as many view the need for sleep as a “weakness” or a way to “harden” military personnel for the purpose of operational effectiveness (3). This perception poses a persistent challenge to the acceptance and successful implementation of effective sleep policies and initiatives within military training, despite strong evidence linking sleep loss to impaired physical, cognitive, and military task performance, alongside increased injury and illness risk among military personnel (2, 8–10). Moreover, these misconceptions about the need for sleep, often ingrained during basic training, may prevent military personnel from self-reporting sleep issues or seeking support before more severe sleep-related health problems develop (3, 11). It should be noted that poor sleep costs the UK economy up to £40 billion annually (1.86% of GDP) (12). While the financial impact of poor sleep on military organizations is less clear, sleep-related health issues likely increase medical consultations, prescription costs, and hospital visits, significantly contributing to healthcare expenses (13).

Evidence demonstrating poor sleep patterns and behaviors in military training would indicate an underappreciation of the importance of sleep within military organizations. The opportunity to achieve minimum sleep duration recommendations is often sacrificed to provide opportunities for more physical and/or military training, or limited due to programme design and scheduling, and behavioral factors, such as lack of education regarding performance and health benefits and decrements associated with good and poor sleep, respectively (14). Additionally, the primary sleep environment in basic training is considered sub-optimal for healthy sleep due to poor sleep hygiene and multi-occupancy rooms (e.g., 12-person bed spaces) which are often used to conduct other non-sleep related activities such as preparation of field-kit, locker inspections, ironing, boot cleaning, socializing and studying (6, 15)—all of which are likely to be key contributors to the self-reported sleep disturbances reported by recruits. Findings from a recent sleep study in British Army recruits during basic training (6) revealed an average sleep duration of < 6 h per night, routine complaints of poor sleep quality and excessive daytime sleepiness, leading to frequent periods of dosing off during daytime military activities. Similarly observed in other military cohorts (1, 7, 15), late-night inspections and military admin, and routine early-morning wake times (~05:30) were reported as the most common factors preventing recruits from achieving sufficient levels of sleep duration, along with excessive light, noise, hot room temperatures, stress and anxiety. As a consequence of these non-sleep promoting conditions, recruits self-reported that their basic training performances (both physical and cognitive) were adversely affected by the chronic sleep loss experienced during basic training.

Acknowledging the implications of these initial observations on recruit health and performance, revisions were made within a subsequent iteration of the British Army's basic training programme, defined as the Common Military Syllabus 21 (CMS21). These revisions proposed a greater opportunity for earlier bedtimes and longer nocturnal sleep periods by capping the working day at 18:30, thus enabling recruits more time to complete their non-scheduled military admin (e.g., kit preparation and inspections, block duties, i.e., cleaning communal areas; studying and self-care, i.e., showering, shaving etc.), that would otherwise be conducted during late evening/night-time hours, thereby compressing the available time to sleep. To determine the efficacy of CMS21 to enable greater sleep opportunity, this study evaluated and compared sleep patterns and perceptions of sleep quality of CMS21 against the prior basic training programme (CMS18). It was hypothesized that recruits undergoing CMS21 would exhibit greater weekly sleep durations and report fewer sleep quality issues compared to their CMS18 counterparts.

## Methods

Full data sets from a total of 93 (CMS18: male = 63; female = 30) and 122 (CMS21: male = 99; female = 23) non-infantry recruits from the Army Training Center, Pirbright [ATC(P)] were used for analysis (Table 1). A sleep ring (OURA Gen 2), width: 7.9 mm; thickness: 2.6 mm; weight: 4–6 g) was worn on the non-dominant index finger and used to record sleep-wake indices each night of the 12-week course (~90 days). Data from the sleep ring was extracted from proprietary software (Oura Teams, Finland) and mean ± SD reported across each week of basic training. Compared to gold-standard (i.e., Polysomnography), the sleep ring demonstrates acceptable levels of accuracy and sensitivity in detecting sleep-wake variables and differences in sleep patterns in young healthy populations (16) along with good levels of acceptability within similar groups of individuals and environments (6). The sleep indices (and units of measure) captured for analysis included, total sleep time [TST, hours:mins (hh:mm)], time in bed (TIB, hh:mm), sleep onset latency (SOL, hh:mm), wake after sleep onset (WASO, hh:mm) and sleep efficiency (SE, %). Definitions of each sleep variable have been reported previously (6).

**Table 1:** Participant demographics.

**Non-infantry recruits**	**Age (yrs)**	**Height (cm)**	**Body mass (kg)**	**BMI (kg/m^2^)**
CMS18 (n 93)	26.2 ± 2.2	172.5 ± 8.8	76.3 ± 3.6	25.6 ± 3.2
CMS21 (n 122)	25.1 ± 1.8	174.9 ± 10.8	78.8 ± 2.1	25.7 ± 2.9

Values are mean ± SD.

Data during CMS18 and CMS21 was collected during spring and autumn, 2021, respectively. Recruits completed a weekly sleep questionnaire measuring perceptions of sleep quality and sleep disturbances. An online version (LimeSurvey, Community Edition Version 6.3.4) of the National Sleep Foundation (NSF) sleep diary (completed on individual's mobile phones) was used to determine perceptions of sleep quality and sleep disturbing factors, involving a series of multiple-choice questions, including “*likeliness of dozing off during daytime*,” “*ease of falling asleep at night*,” “*overall rating of sleep quality*,” “*perceived fatigue upon awakening*,” and “*factors disturbing sleep at night*.” To minimize burden on recruits in training, sleep diary data was only collected during weeks 1, 6 and 12. Sleep questionnaire data was extracted from LimeSurvey and descriptively analyzed in Microsoft Excel (Office 365). Participants were provided with verbal instructions of the timing (i.e., AM between 06:00 and 07:00) and how to complete the self-report measures and were given example question/answer definitions to aid their understanding and interpretation. Ethical approval for CMS18 (924/MODREC/18) and CMS21 (1076/MODREC/20) was granted by the UK Ministry of Defense Research Ethics Committee. Each participants height and weight were recorded during week 1 using a stadiometer and digital weighing scales (SECA 703, Birmingham, UK). Body mass index was calculated and interpreted as per Nuttall (25).

## Statistical analysis

Data was analyzed descriptively (mean ± SD) to summarize participants' demographics, sleep-wake variables and scores for each subjective response across the reporting weeks of basic training. The daily TST derived from the sleep ring was plotted against the NSF recommendations for young adults (18–25 yrs). The R package “vioplot” (17) (V.0.3.6) in R Core Team software (V.4.0.4; Vienna, Austria) was used for the creation of TST, TIB, SOL, WASO and SOL figures. Retrieval of sleep rings was scheduled at the end of week 11 due to scheduling demands during week 12 (i.e., pass off). Sleep-wake variables collected from the sleep ring were graphically examined for normality (normal Q-Q plots) prior to statistical analysis. Sample size was based on *a priori* power analysis using G^*^power (Dusseldorf, V 3.1). For a between-groups repeated-measures design, a minimum of ~80 participants per group was required to detect a medium effect size (np^2^ = 0.06) with α = 0.05 and β = 0.95. Data from the sleep ring was analyzed using a linear mixed model to examine differences in sleep-wake indices between group (CMS18 vs. CMS21) and time (basic training week; alpha set at *p* < 0.05). The model structure included fixed effects for time, group and their interaction, with participant as the random effect to account for individual variability. Significant main effects were followed up with *post-hoc* (Bonferroni adjusted) analyses and mean differences between significant pairs were presented (Supplementary Table 2).

## Results

### Actigraphy sleep-wake indices

**Figure 1:**
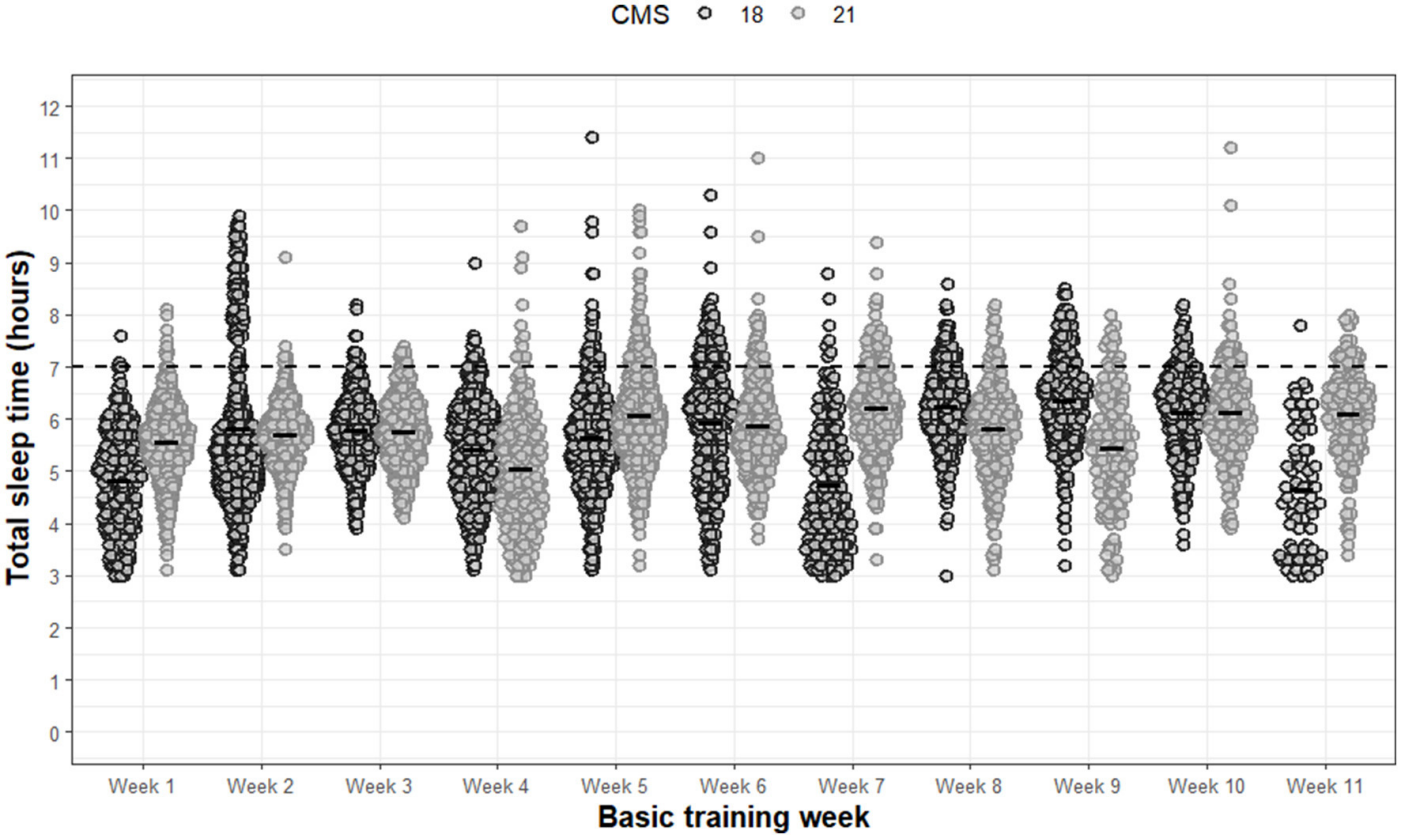
Total sleep time for each night of each week during basic training. Dots are individual nights within their respective week. The solid horizontal lines represent the average weekly total sleep time. The dashed horizontal line represents minimum national sleep duration recommendations for young adults.

Specific to TST (Figure 1), significant main effects of week (F 57.6, *p* < 0.001) and a significant interaction effect between group and week was observed (F 70.1, *p* < 0.001). No significant main effect of group was found for TST (F 3.7, *p* = 0.054). All additional *post-hoc* analyses for TST can be found in Supplementary Table 2. The proportion of weeknights that recruits did not meet minimum sleep recommendations ranged between 67–94% and 69–97% for CMS18 and CMS21, respectively. During ~3-months of basic training, recruits only met national sleep recommendations for 18% to 22% of weeknights in CMS18 and CMS21, respectively. Overall, the average weekly TST for both groups can be defined as inadequate, ranging between 05:11 ± 00:51 hm and 06:07 ± 00:55 hm across basic training.

**Figure 2:**
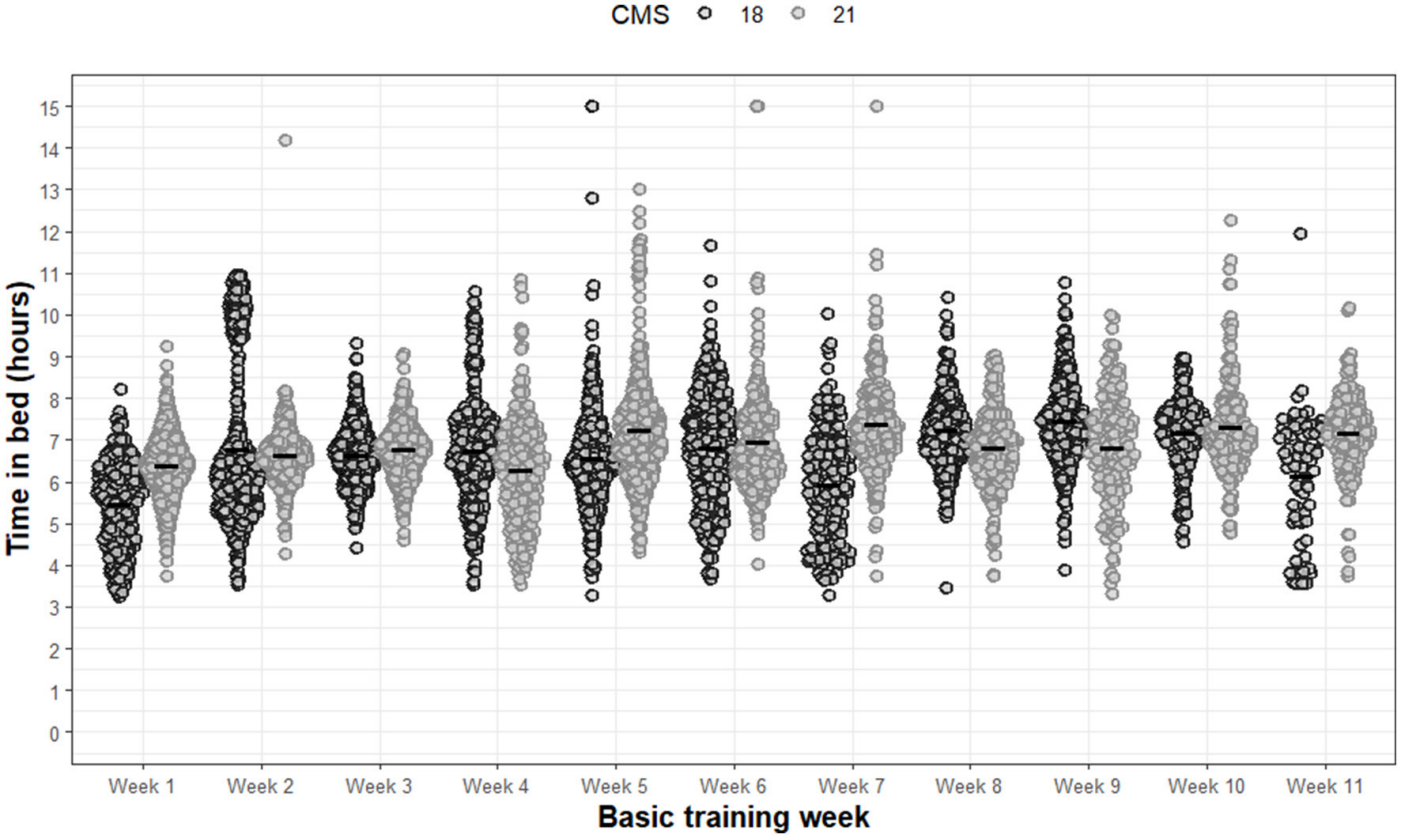
Time in bed for each night of each week during basic training. Dots are individual nights within their respective week. Solid horizontal line represents the average weekly time in bed.

Specific to TIB (Figure 2), significant main effects of group (F 9.8, *p* = 0.002) and week (F 50.8, *p* < 0.001) were found. A significant interaction effect between group and week was also observed (F 43.6, *p* < 0.001). *Post-hoc* analysis revealed that CMS21 demonstrated a significantly greater average TIB compared to CMS18 of 00:13 hm (*p* = 0.002, 95%CI: 0.37–0.08). All additional *post-hoc* analyses for TIB can be found in Supplementary Table 2. Despite slight improvements in the overall average TIB in CMS21, the opportunity for sufficient sleep duration as per national recommendations continued to be limited by inadequate TIB.

**Figure 3:**
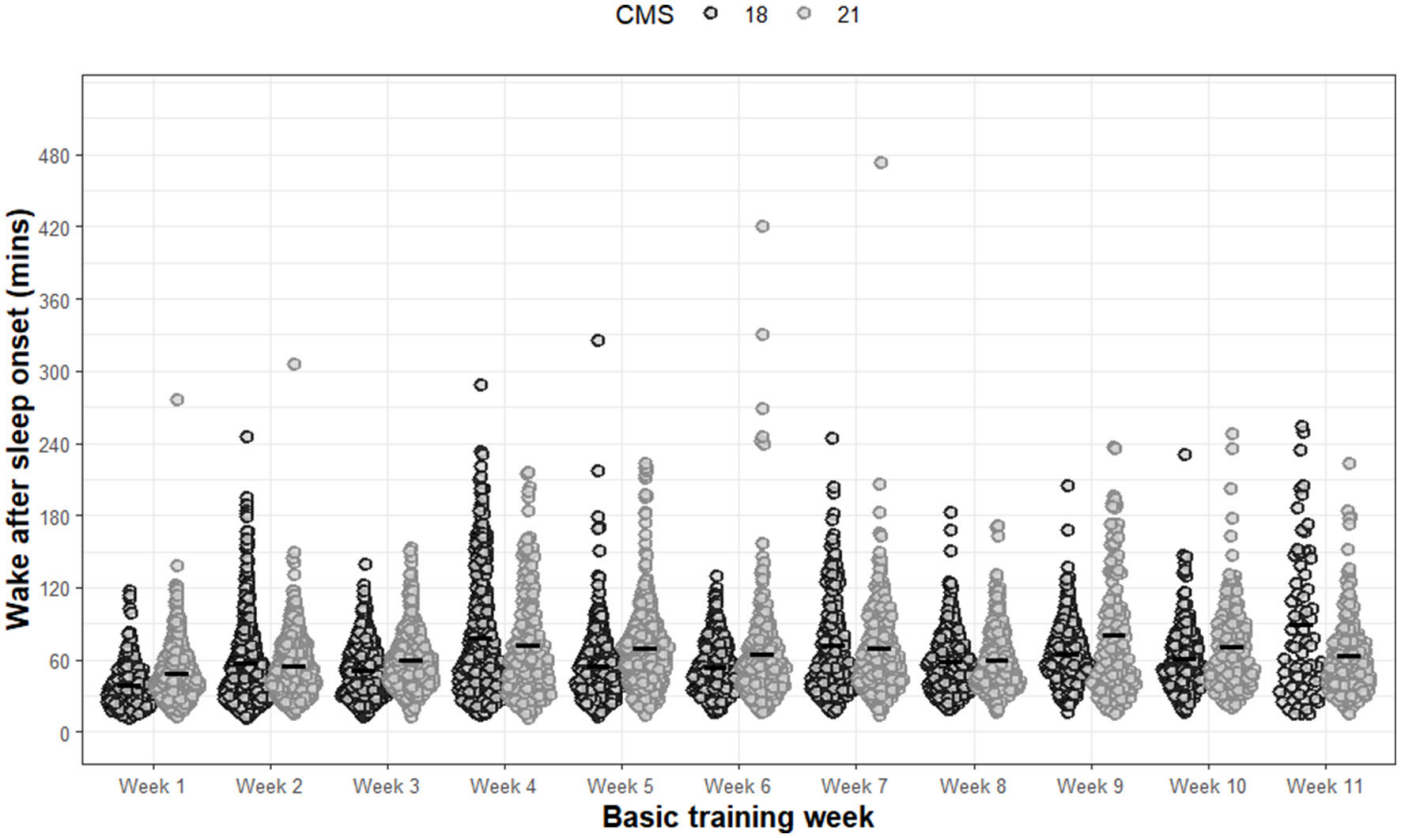
Wake after sleep onset for each night of each week during basic training. Dots are individual nights within their respective week. Solid horizontal line represents the average wake after sleep onset.

For WASO, significant main effects of group (F 5.5, *p* = 0.019) and week (F 53.2, *p* < 0.001) were observed (Figure 3). A significant interaction effect between group and week was also observed (F 12.6, *p* < 0.001). *Post-hoc* analysis revealed that CMS21 demonstrated, on average, significantly greater WASO of 00:07 hm compared to CMS18 (*p* = 0.019, 95%CI: 0.92, 10.2). Additional *post-hoc* analyses specific to WASO are presented in Supplementary Table 2.

**Figure 4:**
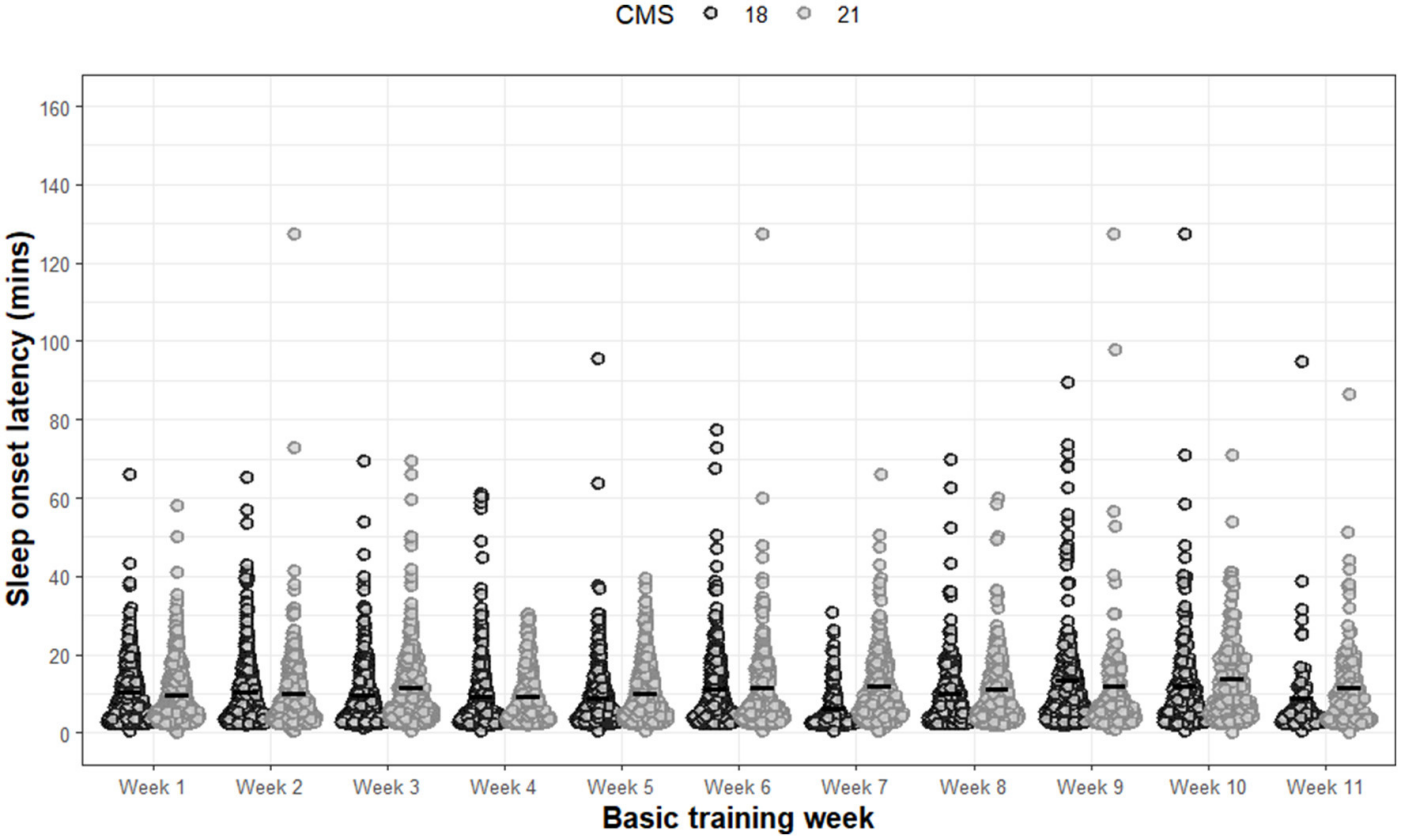
Sleep onset latency for each night of each week during basic training. Dots are individual nights within their respective week. Solid horizontal line represents the average sleep onset latency.

For SOL (Figure 4), significant main effects of week (F 8.01, *p* < 0.001) and significant interaction effects between group and week (F 5.04, *p* < 0.001) were observed. No significant main effects were observed for group (F 1.45, *p* = 0.229). All *post-hoc* analysis for SOL are presented in Supplementary Table 2.

**Figure 5:**
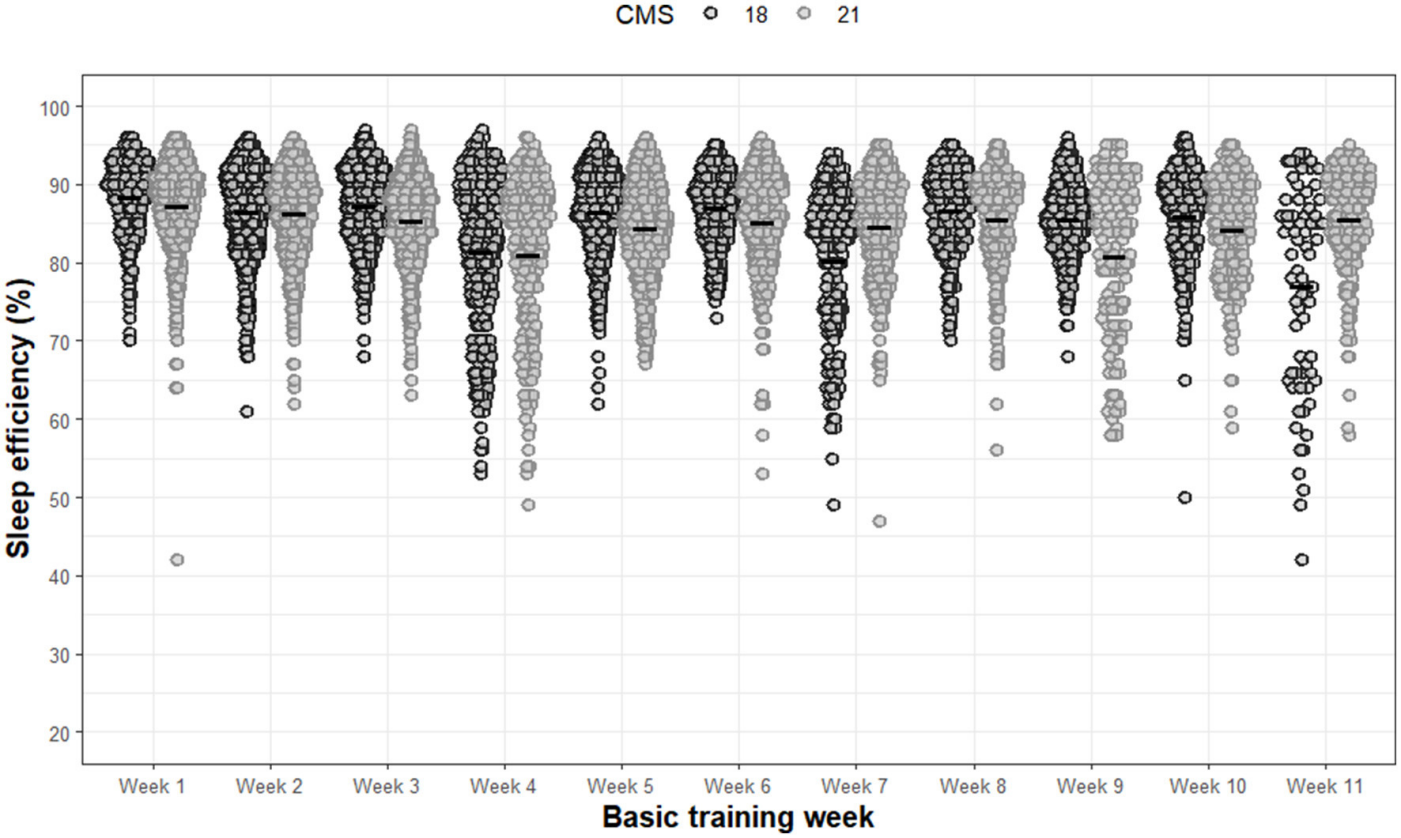
Sleep efficiency for each night of each week during basic training. Dots are individual nights within their respective week. Solid horizontal line represents the average sleep efficiency.

Significant main effects of week (F 72.1, *p* < 0.001) and significant interaction effects between group and week (F 27.1, *p* < 0.001) was observed for sleep efficiency (Figure 5). No significant main effects were observed for group (F 2.5, *p* = 0.122). All *post-hoc* analyses for sleep efficiency are presented in Supplementary Table 2.

### Self-reported sleep outcomes

**Figure 6:**
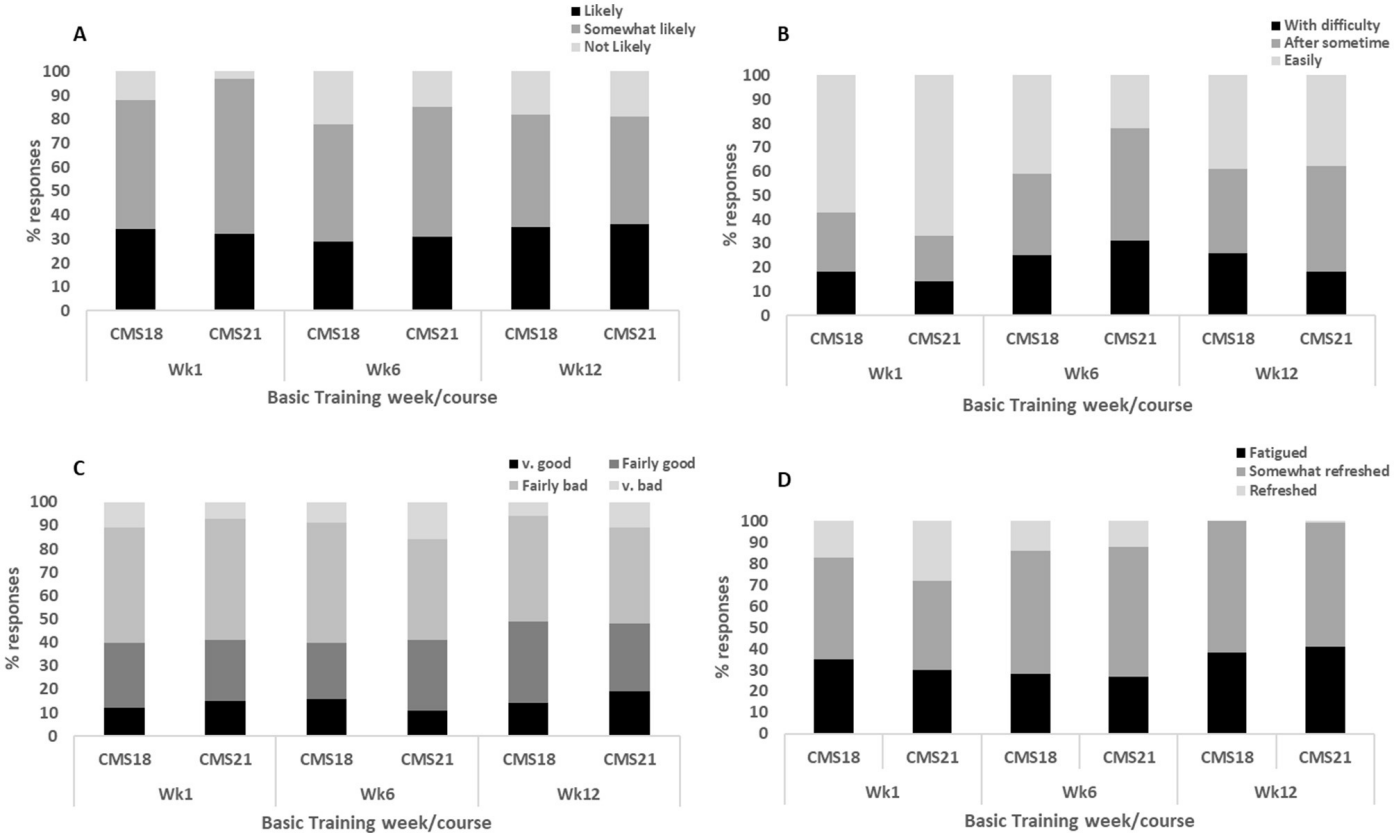
Represents the proportion (%) of responses from recruits for **(A)** “likeliness of dozing off during daytime;” **(B)** “ease of falling asleep at night;” **(C)** “overall rating of sleep quality;” **(D)** “perceived fatigue upon awakening.”

Given the lack of differences in sleep opportunity and similarities in the recruit's sleeping environment, it is unsurprising that across the four sleep diary questions, negligible differences were observed between groups and across the three data collection weeks (Figure 6). Therefore, the range of mean scores across group and week are reported for each self-reported sleep diary question. The majority of responses indicated that recruits in both groups across basic training were “likely” (30–36%) and “somewhat likely” (46–60%) to doze off during the daytime; they rated their sleep quality as “very bad” (9–11%) and “fairly bad” (49–51%), with 24–28% reporting “fairly good” sleep quality; they were able to fall asleep “easily” (32–62%) and “somewhat easily” (22–41%), with 16–28% reporting “difficulties;” and felt “somewhat refreshed” (45–60%) and “fatigued” (28–40%) upon awakening during basic training. The most common self-reported sleep disturbing factor was excessive noise in and around the primary sleeping environment for both CMS18 (32–48%) and CMS21 (22–45%). Other equally common sleep disturbing factors included early morning wake times (37–42%), followed by illness (9–12%; i.e., coughing, sneezing) and feelings of stress and anxiety (9–19%).

## Discussion

Similar to other military populations (10, 18–20), the quality and average sleep duration of recruits has shown to be poor and chronically restricted during British Army basic training (6). In light of these prior observations, revisions were made to CMS21 that restricted daily scheduled training activity from 18:30, thereby providing greater opportunity for earlier bedtimes and longer nocturnal sleep periods during basic training. This study quantified differences in sleep patterns and behaviors relative to these programme changes and compared outcomes against a control group (i.e., CMS18). Overall, significant differences were observed between the two groups for several sleep-wake parameters (Supplementary Table 2). However, CMS21 did not demonstrate any consistent improvements in any actigraphy-derived or self-reported sleep indices compared to CMS18, and on average, did not meet the minimum national sleep duration recommendations for young adults. Therefore, we reject our hypothesis.

The differences observed in the sleep-wake parameters reported in our study can be explained, in part, by the variability within basic training schedules and impact of environmental factors. During week 1, the greater total sleep time during CMS21 compared to CMS18, is consistent with changes in logistics and volume of administrative tasks (i.e., kit collection, haircuts etc) undertaken during the recruit's first week which resulted in longer sleep opportunity. Compared to CMS21, the significantly shorter sleep duration observed during weeks 7 and 11 of CMS18 can be attributed to early-morning tactical (e.g., marksmanship, field exercise) and adventurous training activities, rather than intentional changes in evening sleep opportunity in CMS21. Similarly, field-based tactical training that required early morning wake times was undertaken in week 9 of CMS21, therefore explaining the less time in bed and lower average total sleep times compared to CMS18. It is worth noting that much of the variability in total sleep times observed between groups can be attributed to unscheduled early-morning physical training as to avoid conducting intense exercise in hot environmental temperatures (as per policy, Joint Services Publication [JSP] 375).

During weeks 1, 7, 9 and 11 of basic training, recruits experienced more severe sleep loss compared to the overall average (Δ ~40 min). Although the average sleep duration increased by ~30 min during the succeeding week, in all cases it remained below the minimum national recommendation (i.e., < 7 h) by at least 01:20 hm. These observations indicate a lack of sufficient recovery sleep (commonly referred to as sleep extension) following severe sleep loss despite evidence of enhanced psychomotor, physical and academic performance effects in recruit populations following sleep extension (5, 21, 22). Additionally, chronic short-sleep duration (i.e., < 6 h) has shown to significantly increase the risk of injury (2-fold) (2) and illness (3-fold) (8) in military personnel. In accordance with the outcomes from prior research, our observations highlight the importance of integrating sleep planning (e.g., sleep extension) as a key component of basic training design to offset the significant risk of impaired military performances, injury and illness associated with chronic sleep loss (11).

Despite differences in sleep duration between groups for select weeks of basic training, the overall average morning wake times and bedtimes showed negligible group differences (CMS18 wake times: 05:28 ± 00:56 hm, CMS21: 05:32 ± 00:59 hm; CMS18 bedtimes: 23:32 ± 00:48 hm, CMS21: 23:26 ± 00:33 hm), which is likely a consequence of the variation in weekly bed and wake times, combined with the extent of wake time after sleep onset in both groups across basic training. Nevertheless, our results confirm prior anecdotal observations of a discrepancy between basic training and the expected bed and wake times set in policy (JSP 822), which specifies a bedtime of 22:00 and a wake time of 06:00—despite reveille scheduled for 05:30 each morning. These empirical outcomes warrant review of current sleep-related practices and policies relative to basic training, but also confirms that despite restricting daily scheduled training activity from 18:30, the changes made to CMS21 did not result in earlier bedtimes, nor longer nocturnal sleep periods as intended. Furthermore, given the chronicity of sleep restriction observed in both groups, it is unsurprising that similar proportions of recruits reported poor sleep quality (≤ 51%), excessive daytime sleepiness (e.g., daytime dozing, ≤ 60%), short sleep- onset latency (≤ 62%) and tiredness related to sleep loss upon awakening (≤ 60%) across basic training. Given the frequency of excessive daytime sleepiness observed, the majority of recruits are likely experiencing high levels of sleep propensity, and therefore, it must be questioned as to why, if given the opportunity, were recruits not achieving longer sleep durations during basic training.

Consistent with reports from other military populations (15, 23, 24), the most dominant disturbances reported by recruits in terms of impact on sleep included excessive noise in and around the primary sleeping environment, followed closely by early morning wake times. During this study, the authors noted that most recruits conducted routine military admin [i.e., non-scheduled activity defined as ironing of kit, boot cleaning, revision and general block jobs (e.g., cleaning)] late into the evening within their multi-occupancy dorms (up to 12-persons). Despite restricting scheduled training activity from 18:30 in CMS21, our observations indicate that the current magnitude of non-scheduled training activity, combined with poor sleep hygiene practices (i.e., excessive noise) are key factors contributing to chronic short-sleep duration, and in part, explains the similar bedtimes to that of the control group (CMS18). The current contracted early-morning feeding times have also been identified as a key contributing factor to sleep loss in basic training, necessitating a daily reveille of 05:30 for a 06:00-feeding time which is an hour earlier compared to the expectations set by sleep-related policy (i.e., JSP 882). These scheduling conflicts not only restrict recruits' ability to get sufficient sleep but also likely contribute to sleep deprivation among their training instructors. Instructors must be present to supervise and facilitate activities at both the start and end of each training day, while maintaining a high level of readiness to address emergencies, discipline issues, and meet the individual needs of recruits. Consequently, their ability to effectively fulfill these responsibilities is likely impaired by sleep loss; however, further research is needed to clarify this assertion.

Consistent with prior observations (6), most recruits (~80%) from both groups demonstrated insufficient sleep duration, however, between 16% and 20% were able to achieve minimum recommendations within the constraints of the basic training programme. It remains uncertain how some recruits met the minimum sleep recommendations despite the standardized nature of basic training, the shared sleep environment (e.g., 12-person dorms), and the extent of sleep disturbances identified within the primary sleeping quarters. Whilst no interviews/focus groups were undertaken to elucidate these findings, our observations indicate that some recruits were able to manage their personal military admin (i.e., non-scheduled activity) more effectively than others, and are perhaps more aware of good sleep hygiene practices (e.g., use of ear plugs for noise mitigation). Individuals placed on limited duties due to short-term illness and/or injury may have greater opportunity to achieve longer sleep periods, however, the extent to which injury/illness disrupted (if at all) their sleep was also not measured in this study. Levels of sleep-related education and differences in the cultural perceptions of recruits and training instructors regarding sleep may have additionally contributed to the variability in sleep-wake parameters observed in our study, however, further research is needed to elucidate these claims.

## Conclusion

Despite significant differences between groups for several sleep-wake parameters, the programme modifications of CMS21 failed to demonstrate consistent improvements in measures of sleep duration and quality compared to CMS18, with average total sleep time remaining insufficient relative to the minimum national sleep duration recommendations for young adults. Sleep disturbances, namely excessive noise, early morning wake times, and extensive non-scheduled training activities late into the evening remain key factors contributing to chronic sleep restriction in recruits during basic training (6, 10). However, further investigation is needed to better understand how ~20% of recruits achieved adequate sleep despite these ongoing challenges. The results of this study warrant the application of improved sleep hygiene practices/education within the basic training environment, and it is recommended that the contracted early-morning feeding times and magnitude of non-scheduled training activity be considered as factors for change within future programme design, to provide sufficient opportunity for adequate sleep as stipulated in policy, and by extension, to optimize the many aspects of recruit health and performance by which sleep loss significantly impairs.

## Data Availability

The original contributions presented in the study are included in the article/Supplementary material, further inquiries can be directed to the corresponding author.
